# Preventive Effects of Carnosine on Lipopolysaccharide-induced Lung Injury

**DOI:** 10.1038/srep42813

**Published:** 2017-02-16

**Authors:** Ken-Ichiro Tanaka, Toshifumi Sugizaki, Yuki Kanda, Fumiya Tamura, Tomomi Niino, Masahiro Kawahara

**Affiliations:** 1 Laboratory of Bio-Analytical Chemistry, Research Institute of Pharmaceutical Sciences, Musashino University, 1-1-20 Shinmachi, Nishitokyo-shi, Tokyo 202-8585, Japan; 2 Department of System Chemotherapy and Molecular Sciences, Division of Bioinformatics and Chemical Genomics, Graduate School of Pharmaceutical Sciences, Kyoto University, Sakyo-ku, Kyoto 606-8501, Japan

## Abstract

Acute respiratory distress syndrome (ARDS) is a potentially devastating form of acute lung injury, which involves neutrophilic inflammation and pulmonary cell death. Reactive oxygen species (ROS) play important roles in ARDS development. New compounds for inhibiting the onset and progression of ARDS are required. Carnosine (β-alanyl-L-histidine) is a small di-peptide with numerous activities, including antioxidant effects, metal chelation, proton buffering capacity and the inhibition of protein carbonylation and glycoxidation. We have examined the preventive effects of carnosine on tissue injury, oedema and inflammation in a murine model for ARDS. Oral administration of carnosine suppressed lipopolysaccharide (LPS)-induced vascular permeability, tissue injury and inflammation in the lung. *In vivo* imaging analysis revealed that LPS administration increased the level of ROS and that this increase was inhibited by carnosine administration. Carnosine also suppressed LPS-induced neutrophilic inflammation (evaluated by activation of myeloperoxidase in the lung and increased extracellular DNA in bronchoalveolar lavage fluid). Furthermore, carnosine administration suppressed the LPS-induced endoplasmic reticulum stress response *in vivo*. These results suggest that the oral administration of carnosine suppresses LPS-induced lung injury via carnosine’s ROS-reducing activity. Therefore, carnosine may be beneficial for suppressing the onset and progression of ARDS.

Acute respiratory distress syndrome (ARDS) was first defined in 1967 by the American–European Consensus Conference and is a major clinical problem in intensive care unit patients^1^,^2^. Even though approximately 150,000 individuals per year receive a diagnosis of ARDS in the United States, a standard clinical protocol for treatment has not been established as yet, and ARDS mortality remains at 40–50%^1^,^2^.

ARDS is often associated with pneumonia (direct injury) or sepsis (indirect injury). These conditions result in the pulmonary inflammation and injury that arise from alveolar-capillary membrane damage and leakage of protein-rich oedema fluid into alveoli^3^,^4^,^5^. Epithelial and endothelial damage also induces severe inflammatory responses and an increase in vascular permeability, not only in the lungs but also in other organs, resulting in multiple organ failure^3^,^4^,^5^. Because clinical trials using steroids have not been able to show a significant improvement in patients’ mortality^6^,^7^, steroids are no longer routinely used to treat ARDS patients. Many other pharmacological therapies, such as β-2 agonists, surfactant protein C and statins have been investigated; however, these therapies have not been approved^8^,^9^,^10^,^11^. Therefore, identifying new strategies to prevent ARDS development is very important.

Reactive oxygen species (ROS) have been shown to play a major role in ARDS development^12^,^13^. In ARDS patients, ROS are produced extensively by infiltrating leukocytes, especially neutrophils. ROS damage not only the lung but also other organs, by promoting neutrophilic inflammation, increasing vascular permeability, and activating the coagulation system^12^,^13^. Moreover, ROS induce epithelial and endothelial damage, which are involved in ARDS development^14^. Increases in ROS levels in plasma, expired breath condensates and bronchoalveolar lavage fluid (BALF) have been reported in ARDS patients and ARDS-related animal models^13^,^15^,^16^,^17^,^18^.

ROS are reportedly involved in the formation and development of neutrophilic extracellular traps (NETs)^19^. NETs are composed of decondensed chromatin fibers and cytoplasmic protein, such as myeloperoxidase and neutrophil elastase. NETs are believed to be an important defensive system against bacterial infections. However, excessive activation of NETs is thought to be the cause of various inflammatory diseases, including lung diseases^20^,^21^. Furthermore, NETs formation has also been reported to play a major role in ARDS and a lipopolysaccharide (LPS)-induced lung injury model^20^,^22^,^23^.

The endoplasmic reticulum (ER) stress response results in the accumulation of unfolded and misfolded proteins and is reportedly induced by various stressors, including ROS^24^,^25^. The unfolded protein response (UPR) is an adaptive mechanism to refold unfolded and misfolded proteins in the ER. Glucose-regulated protein 78 (GRP78), a representative ER chaperone, confers protection against stressors by mediating this refolding. However, if ER stress is not resolved by adaptive UPR signaling, ER stress-dependent apoptotic signaling pathways are activated, such as phosphorylation of c-Jun N-terminal kinase and induction of CCAAT/enhancer-binding protein-homologous protein (CHOP)^24^,^25^. Therefore, the ER stress response is implicated in several diseases, such as cancer, inflammatory bowel disease and lung diseases^24^,^26^. Recent studies showed that LPS induced increases in various ER stress markers in the lung, and inhibition of ER stress ameliorated LPS-induced lung inflammation^27^,^28^. Thus, a compound that inhibits neutrophilic inflammation or the ER stress response would suppress the onset and progression of ARDS.

Carnosine (β-alanyl-L-histidine) is a small di-peptide with numerous activities, including antioxidant effects, metal ion chelation, proton buffering capacity, and inhibitory effects on protein carbonylation and glycoxidation^29^,^30^. Carnosine is abundantly present in skeletal muscles, cerebral cortex, kidney, spleen and plasma^30^. We have previously reported that carnosine suppresses Zn^2+^ -induced hypothalamic cell death and the ER stress response^31^,^32^. These results suggested that carnosine protects against Zn^2+^ -induced neurotoxicity by suppressing the ER stress response.

Recently, the efficacy of carnosine against lung injury has been shown in animal models. For example, carnosine ameliorates H9N2 swine influenza virus- or bleomycin-induced lung injury *in vivo*^33^,^34^. Ohata *et al*. shown that polaprezinc, a chelate compound consisting of zinc and carnosine, protects mice against intraperitoneal LPS administration-dependent septic shock^35^. However, the efficacy of carnosine against intratracheal LPS administration-induced lung injury (a major ARDS-related animal model) has not been shown. Furthermore, the protective mechanism of carnosine against LPS-induced lung injury is unknown.

We therefore examined the effect of carnosine in LPS-induced lung injury in the present study. Oral administration of carnosine suppressed oedema, tissue injury and inflammation in the lungs of mice that were administered LPS intratracheally. We also found that administration of carnosine suppressed neutrophilic inflammation (NETs formation) and the ER stress response by decreasing ROS production *in vivo*. These results suggest that carnosine might be beneficial for suppressing the onset and progression of ARDS.

## Results

### Effect of carnosine on LPS-induced lung injury

**Figure 1:**
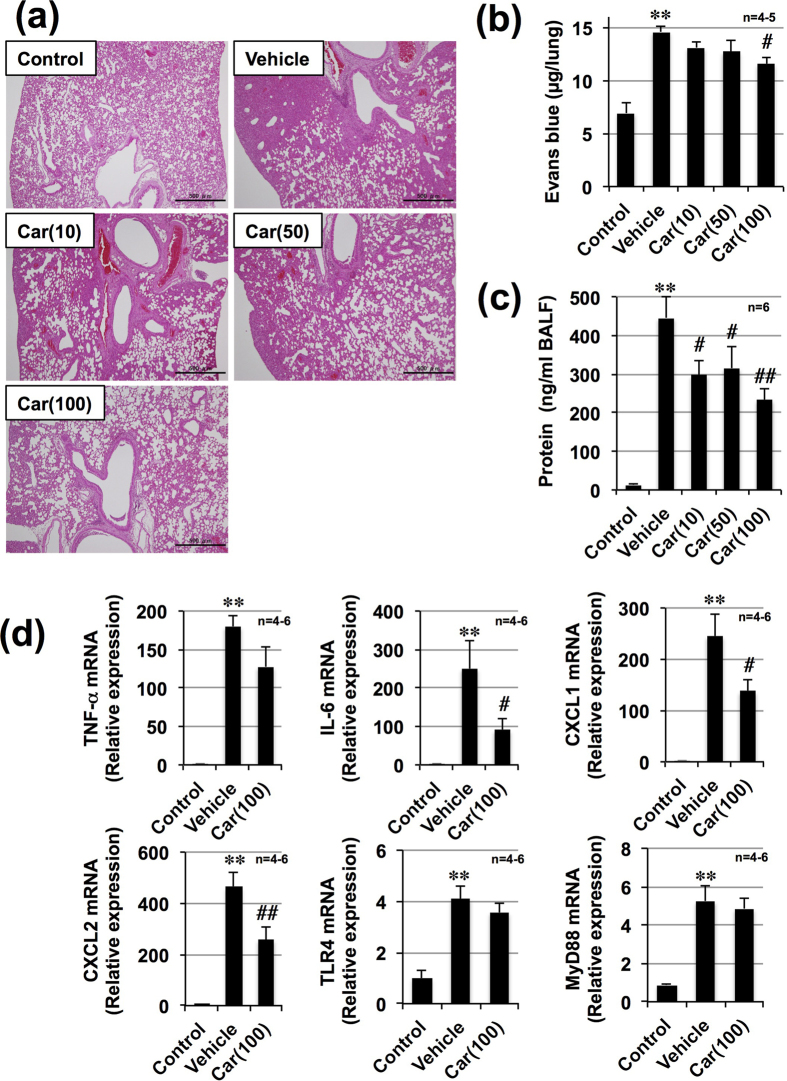
Effect of carnosine on LPS-induced lung injury. Male ICR mice were intratracheally administered with LPS (1 mg/kg) or the LPS vehicle (Control). Mice were orally administered with the indicated doses of carnosine (Car, mg/kg) or saline (Vehicle), immediately before and 24 h after LPS administration (**a,b**), or immediately prior to LPS administration (**c,d**). Sections of pulmonary tissue (**a**) or BALF (**c**) were prepared 48 h after LPS administration. Sections were subjected to histopathological examination (H&E staining) (scale bar, 500 μm) (**a**). Evans blue dye (30 mg/kg) was administered intravenously 6 h after LPS administration, and 2 h later, was extracted from the lung samples and quantified (**b**). The amount of protein present in the BALF was determined by the Bradford method (**c**). Total RNA was extracted from lung 24 h after LPS administration, and subjected to real-time RT-PCR using a specific primer set for each gene. Values were normalized to *Hprt1* and expressed relative to the Control (**d**). Values are mean ± S.E.M.; ^#^*P* < 0.05; ** or ^##^*P* < 0.01 (*, vs Control; #, vs Vehicle).

We examined the effect of carnosine on LPS-induced lung injury, an animal model of acute lung injury. Histopathological analysis of lung sections revealed that intratracheal LPS administration caused alveolar haemorrhage, leukocyte infiltration and severe lung interstitial oedema, and that this injury was suppressed by oral carnosine administration (Fig. 1a). Intratracheal LPS administration increased vascular permeability in lung tissue, and simultaneous carnosine administration suppressed this increase (Fig. 1b). Protein concentration in BALF, an indicator of lung injury and oedema, was also increased by LPS administration, and carnosine administration suppressed this increase (Fig. 1c). Moreover, LPS administration induced expression of pro-inflammatory cytokines (tumour necrosis factor-α, TNF-α and interleukin-6, IL-6) and chemokines (chemokine (CXC motif) ligand-1, CXCL1, and CXCL2) in lung tissue, whereas carnosine administration significantly suppressed this increase, except for TNF-α (Fig. 1d). In contrast, carnosine did not affect the LPS-induced expression of Toll-like receptor 4 (TLR4) and myeloid differentiation primary response gene 88 (MyD88). Overall, the results shown in Fig. 1 suggest that oral carnosine administration protects mice against LPS-induced lung injury.

In addition to the LPS-induced lung injury model, we also examined the effect of carnosine in a zymosan (a TLR2 ligand)-induced lung injury model. As shown in Supplementary Fig. S1, zymosan administration induced lung injury, increasing the protein concentration and the number of neutrophils in BALF. These effects were suppressed by the oral carnosine administration.

### Effect of carnosine on LPS-induced ROS increase *in vivo*

**Figure 2:**
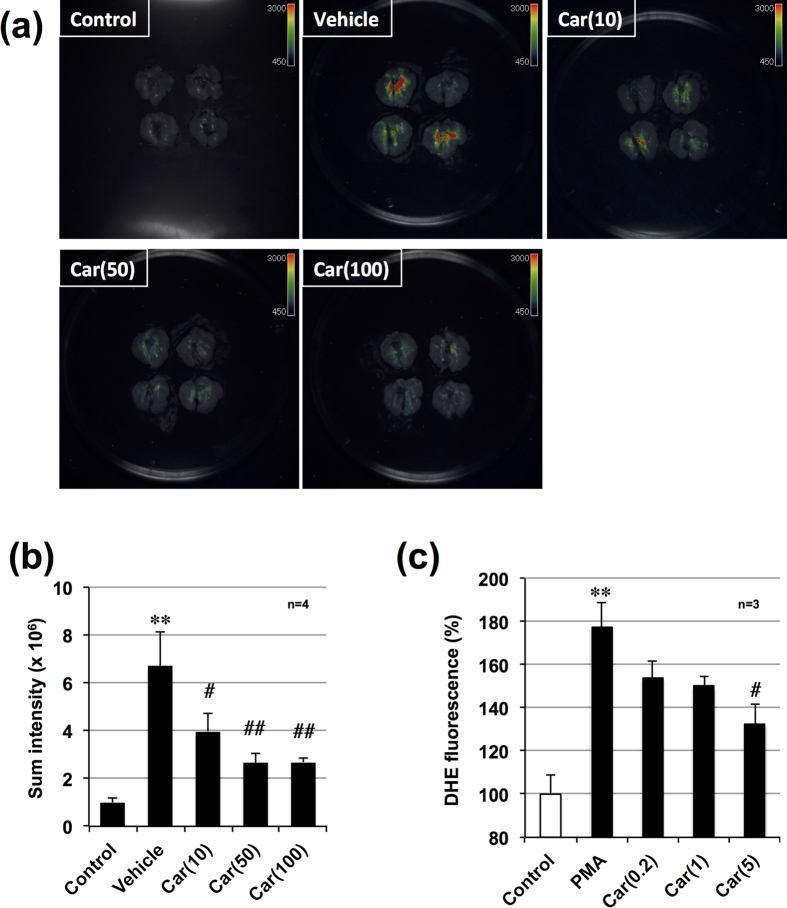
Effect of carnosine on the level of ROS *in vivo* and *in vitro*. Male ICR mice were intratracheally administered with LPS (1 mg/kg) or the LPS vehicle (Control). Mice were orally administered with the indicated doses of carnosine (Car, mg/kg) or saline (Vehicle) immediately prior to LPS administration. Luminescent probe (L-012, 75 mg/kg) was administered 6 h after the LPS administration. Isolated lungs were imaged using a Lumazone *in vivo* imaging system (**a**). The summed pixel intensity of the ROS signal was determined using SlideBook 6 software (**b**). RAW264 cells were pre-incubated with the indicated concentrations of carnosine (Car, mM) for 30 min. They were then incubated with PMA (100 nM) for 30 min in the presence of dihydroethidium (DHE), an indicator of superoxide. DHE fluorescence was measured using a fluorescence microplate reader (**c**). Values represent mean ± S.E.M. ^#^*P* < 0.05; ** or ^##^*P* < 0.01 (b: vs Control; #, vs Vehicle, c: * vs Control, # vs PMA).

ROS are involved in the onset and progression of acute lung injury^12^,^13^. We therefore monitored ROS levels using an *in vivo* imaging system. As shown in Fig. 2a and b, intratracheal LPS administration increased ROS levels in the lung. Conversely, simultaneous carnosine administration clearly suppressed this LPS-dependent ROS increase. We further examined the ROS-reducing activity of carnosine *in vitro*. As shown in Fig. 2c, treatment of RAW264 cells with phorbol myristic acid (PMA) induced ROS production. Addition of carnosine significantly suppressed the production of ROS. These results suggest that carnosine suppressed LPS-induced lung injury by suppressing increases in ROS levels.

### Effect of carnosine on LPS-induced inflammatory responses

**Figure 3:**
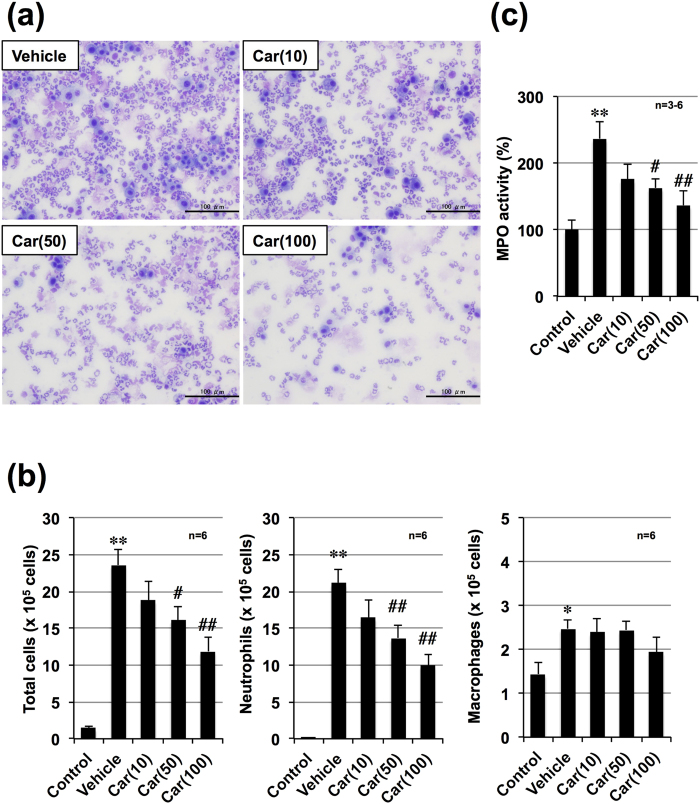
Effect of carnosine on LPS-induced neutrophilic inflammation. Male ICR mice were intratracheally administered with LPS (1 mg/kg) or LPS vehicle (Control). Mice were orally administered with the indicated doses of carnosine (Car, mg/kg) or saline (Vehicle), immediately before and 24 h after LPS administration. BALF and lung homogenates were prepared 48 h after LPS administration. BALF cells were stained with Diff-Quik reagents after centrifugation with a Cytospin^®^ 4 (scale bar, 100 μm) (**a**). The total cell numbers and the neutrophil numbers were determined (**b**). MPO activity in lung homogenates was measured using an MPO assay kit according to the manufacturer’s protocol (**c**). Values are mean ± S.E.M.; * or ^#^*P* < 0.05; ** or ^##^*P* < 0.01. (*, vs Control; #, vs Vehicle).

ROS are an exacerbating factor in neutrophilic inflammation^19^,^36^. We therefore monitored LPS-induced neutrophilic inflammatory responses by measuring the number of leucocytes in BALF 48 h after LPS administration. As shown in Fig. 3a,b, the total number of leucocytes, and especially the number of neutrophils, was increased by the LPS treatment, and these effects were suppressed by simultaneous carnosine administration. In contrast, LPS administration slightly increased the number of macrophages, and these increases were not suppressed by carnosine administration. We also examined the effect of carnosine on myeloperoxidase (MPO) activity (a marker of neutrophilic inflammation) in lung tissues. As shown in Fig. 3c, MPO activity in lung tissues was increased by LPS administration and this increase was suppressed by oral carnosine administration. Moreover, as shown in Supplementary Fig. S2, LPS treatment increased neutrophil elastase-positive cells in lung tissue, and this increase was clearly suppressed by carnosine administration.

**Figure 4:**
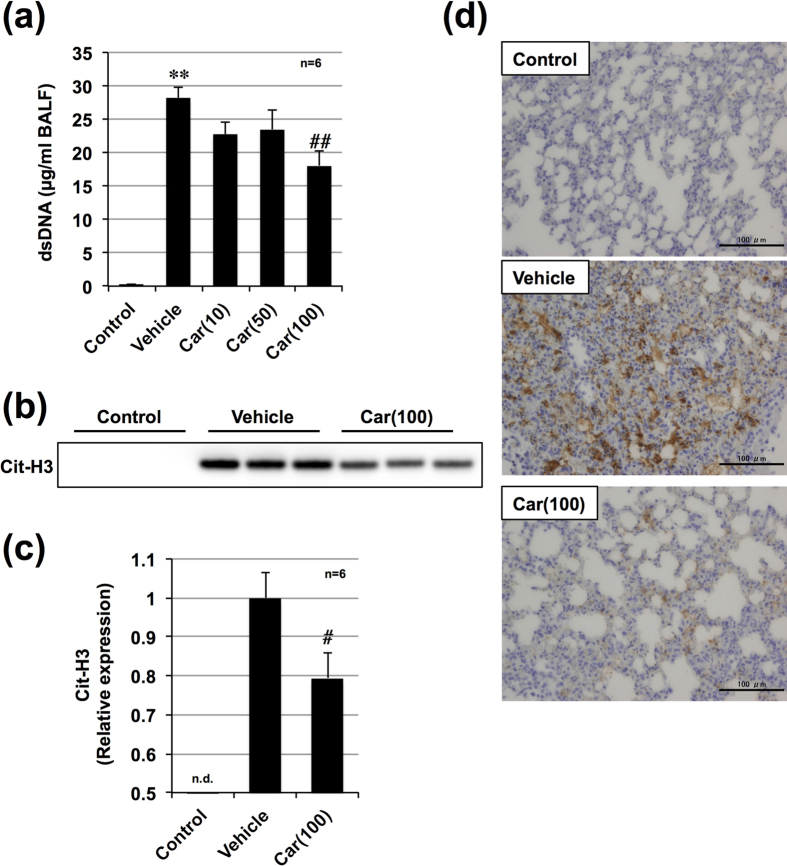
Effect of carnosine on LPS-induced neutrophil extracellular trap formation. Male ICR mice were intratracheally administered with LPS (1 mg/kg) or LPS vehicle (Control). Mice were orally administered with the indicated doses of carnosine (Car, mg/kg) or saline (Vehicle), immediately before and 24 h after LPS administration. BALF (**a–c**) and sections of pulmonary tissue (**d**) were prepared 48 h after LPS administration. The amount of double-stranded DNA (dsDNA) present in the BALF was determined using the Quant-iT™ PicoGreen^®^ dsDNA Reagent and Kits according to the manufacturer’s protocol (**a**). BALF samples (2 μL) were analyzed by immunoblotting with an antibody against citrullinated histone H3 (Cit-H3) (**b**). The Cit-H3 band intensity was determined using Image J software. In the Control group, Cit-H3 expression was not detected (n.d.) (**c**). Immunohistochemical analysis of pulmonary tissue with an antibody against Cit-H3 was performed (scale bar, 100 μm) (**d**). Values are mean ± S.E.M.; ^#^*P* < 0.05; ** or ^##^*P* < 0.01 (*, vs Control; #, vs Vehicle).

Neutrophils release their DNA into the extracellular space to produce NETs^20^,^22^,^23^. As indicators of NETs formation, we monitored dsDNA levels in BALF, and citrullinated histone H3 (Cit-H3) levels in BALF and lung sections. As shown in Fig. 4a, dsDNA levels were increased by the LPS treatment and suppressed by simultaneous carnosine administration. The expression of Cit-H3 in BALF was also induced by the LPS treatment, and was suppressed by carnosine administration (Fig. 4b,c). Moreover, the expression of Cit-H3 in lung tissue was induced by LPS treatment, and again was clearly suppressed by carnosine administration (Fig. 4d). The results in Figs 3 and 4 suggest that carnosine administration protects against LPS-induced lung injury by suppressing neutrophilic inflammation.

### Effect of carnosine on LPS-induced pulmonary cell death and the ER stress response

**Figure 5:**
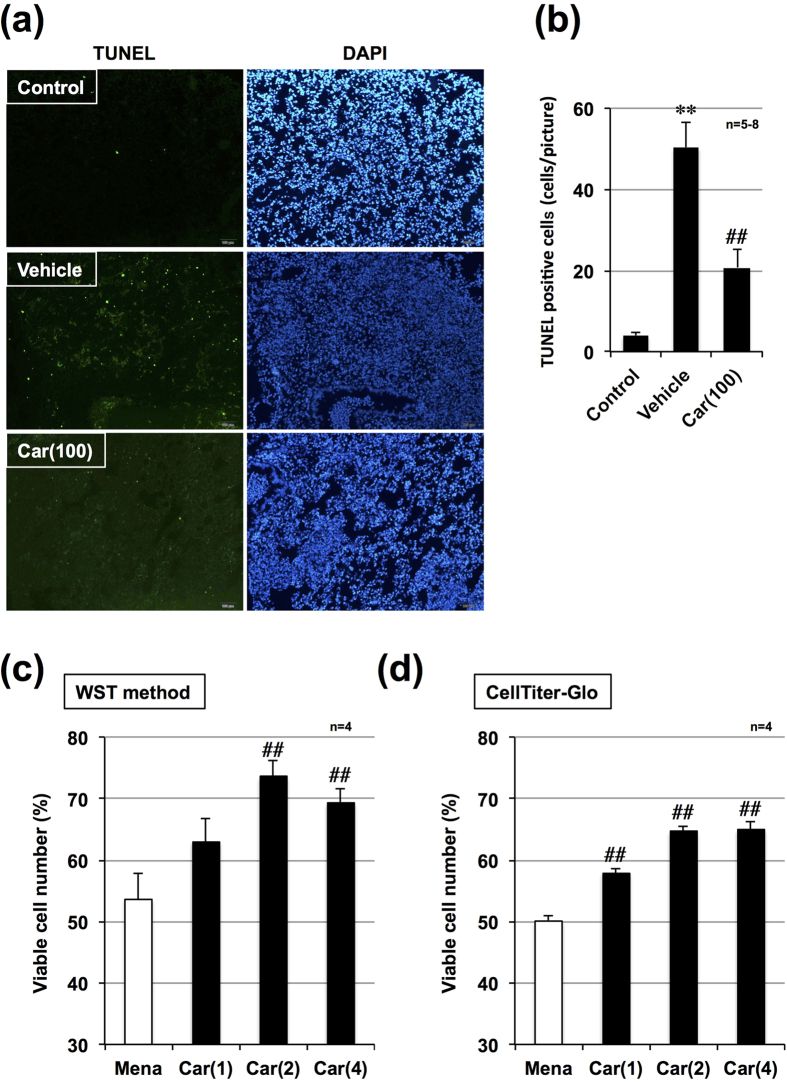
Effect of carnosine on LPS-induced pulmonary cell death. Male ICR mice were intratracheally administered with LPS (1 mg/kg) or LPS vehicle (Control). Mice were orally administered with carnosine (Car; 100 mg/kg) or saline (Vehicle), immediately before and 24 h after LPS administration. Sections of pulmonary tissue were prepared 48 h after LPS administration. Sections were subjected to the TUNEL assay and DAPI staining (scale bar, 100 μm) (**a**). The numbers of TUNEL-positive cells were counted (**b**). A549 cells were incubated with menadione (Mena, 4 μM) for 24 h in the presence of the indicated concentrations (mM) of carnosine (Car). Viable cell numbers were determined using a WST-based cell counting kit (**c**) or CellTiter-Glo^®^ 2.0 (**d**). Values are mean ± S.E.M.; ** or ^##^*P* < 0.01 (**b**: *, vs Control; #, vs Vehicle, **c,d**: # vs menadione).

ROS are thought to induce pulmonary cell death^14^. We therefore examined the effect of carnosine on LPS-induced pulmonary cell death using a terminal deoxynucleotidyl transferase dUTP nick-end labelling (TUNEL) assay. As shown in Fig. 5a,b, intratracheal LPS administration increased TUNEL-positive cells in the lung. Conversery, simultaneous carnosine administration suppressed LPS-induced pulmonary cell death. Next, we examined the cytoprotective effects of carnosine *in vitro*. As shown in Fig. 5c,d, treatment of A549 cells with menadione (to induce oxidative stress) decreased viable cell numbers.

**Figure 6:**
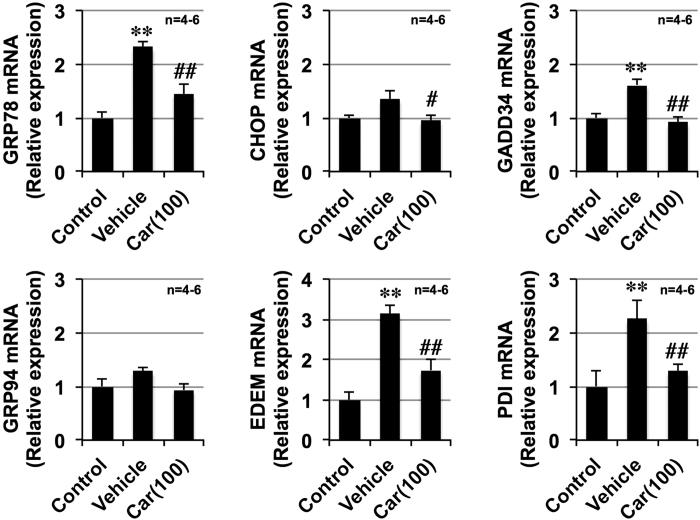
Effect of carnosine on the LPS-induced ER stress response. Male ICR mice were intratracheally administered with LPS (1 mg/kg) or LPS vehicle (Control). Mice were orally administered with carnosine (Car; 100 mg/kg) or saline (Vehicle) immediately before LPS administration. Total RNA was extracted from lung and subjected to real-time RT-PCR using a specific primer set for each gene, 24 h after LPS administration. Values were normalized to *Hprt1* and expressed relative to the Control. Values are mean ± S.E.M.; ^#^*P* < 0.05; ** or ^##^*P* < 0.01 (*, vs Control; #, vs Vehicle).

Carnosine is thought to suppress the ER stress response^31^. Therefore, we monitored the expression of ER stress-related genes in lung tissue using real-time RT-PCR. LPS treatment induced the expression of *Grp78, Gadd34, Edem and Pdi* mRNA; carnosine administration significantly suppressed the expression of *Grp78, Chop, Gadd34, Edem and Pdi* mRNA (Fig. 6). These results suggest that carnosine administration protects against LPS-induced pulmonary cell death by suppressing the ER stress response.

## Discussion

Carnosine is abundantly present in muscle and brain, and has various beneficial effects such as antioxidant activity, metal chelating effects, proton buffering capacity, anti-tumour cell growth activity and the inhibition of protein carbonylation and glycoxidation. Hence, carnosine is thought to be a good candidate for anti-aging or neuroprotective therapy^37^,^38^,^39^. Recently, we reported that carnosine suppresses Zn^2+^ -induced neuronal cell death, suggesting that carnosine could be a candidate for preventing or treating vascular-type dementia^31^,^32^. In the present study, we were interested in examining the effects of carnosine on ARDS development using an LPS-induced lung injury model. We showed that oral carnosine administration suppresses LPS-induced lung injury and inflammation, and we propose that carnosine may be beneficial for preventing ARDS development.

We focused on neutrophilic inflammation and the ER stress response as mechanisms of carnosine’s preventive effect on ARDS development. As shown in Figs 3 and 4, carnosine administration inhibited LPS-induced neutrophilic inflammation. Furthermore, as shown in Figs 5 and 6, carnosine administration inhibited the LPS-dependent ER stress response. ROS are reportedly thought to induce neutrophilic inflammation and the ER stress response^19^,^24^,^26^,^36^. For example, extracellular superoxide induces NETs, and treatment with diphenyleneiodonium, an inhibitor of NADPH oxidase, inhibits this NETs induction^40^. Regarding ER stress, cigarette smoke extract induces the apoptosis of bronchial epithelial cells through a superoxide anion-triggered signaling pathway mediated by protein kinase RNA-like endoplasmic reticulum kinase–eukaryotic initiation factor 2 alpha kinase (PERK-eIF2a, one of the ER stress sensors)^41^. We showed that carnosine administration suppressed the LPS-dependent ROS increase in lung tissue (Fig. 2). Considering these results, we suggest that carnosine suppressed both neutrophilic inflammation and the ER stress response by suppressing LPS-dependent ROS increases.

ARDS is often associated with not only pneumonia but also sepsis, and caecal puncture ligation (CLP) in mice induces phenomena similar to those observed in ARDS patients^42^. Moreover, mechanical ventilation (MV), a life-saving intervention for ARDS patients, also causes ventilator-induced lung injury (VILI), which increases mortality^43^. ROS have been shown to play a major role in sepsis and VILI in both clinical and animal models. In contrast, carnosine administration clearly suppressed the LPS-dependent ROS increase (Fig. 2). Although we have not examined the effects of carnosine in mice subjected to CLP or MV, we assume that carnosine administration may have preventive effects against CLP- or MV-induced lung injury by suppressing the associated ROS increases.

Considering the clinical application of carnosine, it is important to examine the effect of carnosine after the onset of lung injury, and the effect of carnosine on prognosis. As shown in Supplementary Fig. S3, even when carnosine was administered after LPS, carnosine suppressed the LPS-induced increase in the number of neutrophils and the protein concentration in BALF. Intratracheal administration of LPS and hydrochloric acid, a newly reported ARDS-related model^44^, as shown in Supplementary Fig. S4, this was lethal to mice, but carnosine administration increased their survival rate (P = 0.036). These results support our view that carnosine has the beneficial effects for preventing ARDS development.

We found that oral carnosine administration clearly suppressed LPS-induced ROS increases. To our knowledge, this is the first time that the ROS-reducing activity of carnosine has been confirmed by *in vivo* imaging analysis. However, the mechanism by which carnosine administration suppressed ROS production needs to be clarified. Carnosine treatment was previously reported to induce copper/zinc superoxide dismutase (Cu/Zn-SOD) and glutathione peroxidase enzymatic activities in a rat experimental subarachnoid haemorrhage model^45^. Furthermore, other groups showed that carnosine has a hydroxyl radical scavenging effect^46^,^47^. We suggest that carnosine suppresses LPS-dependent ROS increases by these mechanisms.

The effects of other antioxidant molecules on preventing ARDS have already been reported. For example, Cu/Zn-SOD or lecithinized Cu/Zn-SOD administration is also protective in ARDS-related animal models^48^,^49^. The new vitamin E derivative, ETS-GS, protected against CLP-induced systemic inflammation in rats^50^. Early administration of lipoic acid provided protective effects against LPS-induced oxidative stress in the lung^51^. Moreover, simvastatin reduced LPS-induced lung injury by decreasing neutrophil recruitment and radical formation^52^. Some investigations suggested beneficial effects of statins therapy in patients with sepsis and ARDS^53^,^54^, although stains have not been approved for this use. Therefore, it is necessary to compare the protective effects of carnosine against LPS-induced lung injury with those of other antioxidant molecules. Furthermore, combination effects of other antioxidants with carnosine have been reported in other organs. For example, carnosine plus vitamin E treatment more strongly suppressed LPS-induced liver injury compared with carnosine or vitamin E alone^55^. Moreover, α-lipoic acid and carnosine supplementation increased antioxidant activity in the serum, liver and skin of rats^56^. Examining combination effects of other antioxidants with carnosine in the LPS-induced lung injury model would be highly worthwhile.

In conclusion, we revealed that oral administration of carnosine suppresses LPS-dependent lung injury and inflammation. These results suggest that carnosine may be beneficial for suppressing the onset and progression of ARDS. Moreover, carnosine is used in anti-aging supplements in the United States. Therefore, carnosine could be useful not only as a prophylactic drug but also as a supplement preventing the onset and progression of ARDS.

## Materials and Methods

### Chemicals and animals

LPS from *Escherichia coli* (055:B5) was obtained from Sigma (St. Louis, MO). Diff-Quik was from Sysmex (Kobe, Japan). An antibody against actin was purchased from Santa Cruz Biotechnology (Santa Cruz, CA). Antibodies against histone H3 (citrulline R2 + R8 + R17) and neutrophil elastase were purchased from Abcam (Cambridge, UK). L-carnosine, luminal-based chemiluminescent probe (L-012), Evans Blue Dye, zymosan, isoflurane and formalin neutral buffer solution were from WAKO Pure Chemicals (Tokyo, Japan). 4,6-diamidino-2-phenylindole (DAPI) was purchased from Dojindo (Kumamoto, Japan). RNeasy^®^ kit was obtained from Qiagen (Hilden, Germany), PrimeScript^®^ 1^st^ strand cDNA Synthesis Kit was from Takara Bio (Ohtsu, Japan), and SsoFast™ EvaGreen Supermix was from Bio-Rad (Hercules, CA). Mounting medium for immunohistochemical analysis (VECTASHIELD™) was purchased from Vector Laboratories (Burlingame, CA). Mayer’s haematoxylin, 1% eosin alcohol solution and mounting medium for histological examination (malinol) were from MUTO Pure Chemicals (Tokyo, Japan). Novo-Heparin (5000 units) for injection was from Mochida Pharmaceutical (Tokyo, Japan). A549 cells or RAW264 cells were purchased from the American Type Culture Collection (Manassas, VA) or RIKEN BioResource Center (Tsukuba, Japan). ICR mice (6–7 weeks old, male) were purchased from Charles River (Yokohama, Japan). The experiments and procedures described here were carried out in accordance with the Guide for the Care and Use of Laboratory Animals as adopted and promulgated by the National Institutes of Health, and were approved by the Animal Care Committee of Musashino University.

### Treatment of mice with LPS and carnosine

Mice anesthetized with isoflurane were given a single intratracheal injection of LPS (1 mg/kg) in 0.9% NaCl (2 ml/kg), zymosan (1 mg/kg) in 0.9% NaCl (2 ml/kg) or 0.2 M hydrochloric acid (2 ml/kg) using a P200 micropipette via the mouth. During administration, the nostrils of the mice were blocked with a finger, so that the solutions were inhaled from the mouth into the respiratory tract as the mice breathed.

Mice were orally administered carnosine (10, 50, 100 mg/kg) in 0.9% NaCl by syringe using a sonde needle. The first administration of carnosine was given immediately before LPS administration (except for Supplementary Fig. S3). In control experiments, we examined the effect of administering carnosine alone, and found that it did not affect the lung histology, the protein concentrations or number of leukocytes in BALF, the plasma levels of malondialdehyde (an indicator of ROS), or the number of neutrophils in blood (Supplementary Fig. S5). The levels of malondialdehyde in the plasma were determined using TBARS Assay Kit (Cayman Chemical, Ann Arbor, MI). Measurement of neutrophil number was performed by the LSI Medience Corporation Central Laboratory (Tokyo, Japan), using a Sysmex XT-2000iV™ automated haematology analyser (Sysmex, Kobe, Japan).

### Evaluation of lung permeability

To quantitatively examine lung permeability, Evans blue dye (30 mg/kg) was intravenously administered 2 h before the mice were sacrificed. Tissue samples were cut into pieces and incubated with formamide solution at 60 °C for 24 h. Samples were centrifuged to obtain supernatants, the absorbances of which were measured at 620 nm to determine the amount of Evans blue dye present.

### Preparation of BALF

BALF was collected by cannulating the trachea and lavaging the lung twice with 1 ml of sterile 0.9% NaCl containing 50 units/ml heparin. Approximately 1.8 ml of BALF was routinely recovered from each mouse and the total cell number was counted using a hemocytometer. After centrifugation with a Cytospin^®^4 (Thermo Fisher Scientific, Waltham, MA), cells were stained with Diff-Quik reagents and the ratio of neutrophils to total cell number was determined. The amount of protein and double-stranded DNA (dsDNA) present in the BALF was evaluated by the Bradford method and by using a Quant-iT™ PicoGreen^®^ dsDNA Assay Kit (Thermo Fisher Scientific).

### Measurement of ROS by *in vivo* imaging analysis

*In vivo* imaging of ROS in mice was performed as described previously^49^,^57^, with some modifications. We used an imaging system (Lumazone *in vivo* imaging system; Shoshin Em, Okazaki, Japan), which contains a chamber equipped with an electron-multiplying CCD camera. Mice were intravenously administered with the luminescent probe, L-012, in saline (75 mg/kg). At 5 min after the L-012 injection, mice were euthanized and the lungs were rapidly dissected and imaged (5 min exposure). All data were analyzed using SlideBook 6 software (Intelligent Imaging Innovations, Inc., Denver, CO).

### Real-time reverse transcription (RT) polymerase chain reaction (PCR) analysis

Total RNA was extracted from lung tissue using an RNeasy kit according to the manufacturer’s protocol. Samples were reverse-transcribed using the PrimeScript^®^ kit described above. The synthesized cDNA was used in real-time PCR experiments with SsoFast EvaGreen Supermix and analyzed with a Bio-Rad (Hercules, CA) CFX96™ real-time system and CFX Manager™ software. Specificity was confirmed by electrophoretic analysis of reaction products and by the inclusion of template- or reverse transcriptase-free controls. To normalize the amount of total RNA present in each reaction, hypoxanthine phosphoribosyltransferase 1 (HPRT1) cDNA was used as an internal standard. Primers were designed using Primer3 or Primer-BLAST websites. Primers sequences will be provided upon request.

### Immunoblotting analysis and measurement of MPO activity

BALF or homogenized lung samples were prepared. BALF samples (2 μL) were then applied to NuPAGE^®^ Novex 4–12% Bis-Tris Gel (Thermo Fisher Scientific) and subjected to electrophoresis. After western blotting, proteins were detected with their respective antibodies and chemiluminescent staining using SuperSignal™ West Dura Extended Duration Substrate (Thermo Fisher Scientific). Band intensities were quantitated by using ImageJ software (version 1.39 u). The MPO activity of homogenized lung samples was measured using an MPO assay kit (BioVision, Milpitas, CA) according to the manufacturer’s protocol.

### Histological and immunohistochemical analyses and TUNEL assay

Tissue samples were fixed in 10% neutral buffered formalin for 24 h, and then embedded in paraffin before being cut into 4 μm thick sections. Sections were stained first with Mayer’s hematoxylin and then with 1% alcoholic eosin (H&E staining). Slides were mounted with malinol and visualized with a microscope and digital camera (Olympus DP71; Tokyo, Japan).

For immunohistochemical analysis, sections were incubated with Tris–EDTA buffer (pH 9.0) or proteinase K (20 μg/ml) for antigen retrieval and then incubated with DAKO^®^ peroxidase blocking reagent for removal of endogenous peroxidase activity. Sections were blocked with 3% goat serum or 5% goat serum for 10 min, incubated overnight with rabbit anti-histone H3 antibody (1:100 dilution) or for 2 h with rabit anti-neutrophil elastase antibody (1:100 dilution) in DAKO^®^ Antibody Diluent, and then incubated with a DAKO^®^ EnVision peroxidase-labelled polymer–goat anti-rabbit immunoglobulin conjugate for 1 h. Then, 3,3′-diaminobenzidine was applied to the sections for colour development. The sections were finally counterstained with Mayer’s haematoxylin. Slides were mounted with malinol and visualized with a microscope and digital camera (Olympus DP71; Tokyo, Japan).

For the TUNEL assay, sections were incubated first with proteinase K (20 μg/ml) for 15 min at 37 °C, then with terminal deoxynucleotide transferase and biotin-14-ATP for 1 h at 37 °C, and finally with an Alexa Fluor 488–streptavidin conjugate and DAPI (5 μg/ml) for 2 h. Slides were mounted with Vectashield and inspected with the aid of a microscope and digital camera (Olympus DP71).

### Cell culture

A549 cells (a human type II pulmonary epithelial cell line) and RAW264 cells (a mouse macrophage-like cell line) were cultured in Dulbecco’s modified Eagle’s medium supplemented with 10% fetal bovine serum. All cells were cultured in a humidified atmosphere of 95% air with 5% CO_2_ at 37 °C. Viable cell number was quantified using a WST-based cell counting kit (Dojindo, Kumamoto, Japan) or CellTiter-Glo^®^ 2.0 (Promega Corporation, Madison, WI). The levels of ROS *in vitro* were quantified using dihydroethidium (DHE), an indicator of superoxide (Thermo Fisher Scientific).

### Statistical analysis

All values are expressed as the mean ± S.E.M. Two-way ANOVA followed by Dunnett’s test or the Student’s *t*-test for unpaired results was used to evaluate differences between three or more groups or between two groups, respectively. Kaplan–Meier plots were used to describe survival data and log-rank tests was performed to assess statistical differences. SPSS24 software was used for all statistical analyses. Differences were considered to be significant for values of *P* < 0.05.

## Additional Information

**How to cite this article:** Tanaka, K.-I. *et al*. Preventive Effects of Carnosine on Lipopolysaccharide-induced Lung Injury. *Sci. Rep.* **7**, 42813; doi: 10.1038/srep42813 (2017).

**Publisher's note:** Springer Nature remains neutral with regard to jurisdictional claims in published maps and institutional affiliations.

## Supplementary Material

Supplementary File
