# Supplementary material for: Preventive Effects of Carnosine on Lipopolysaccharide-induced Lung Injury
Source: Sci Rep. 2017 Feb 16;7:42813. doi: 10.1038/srep42813 (PMC5311717; doi:10.1038/srep42813)
Supplement: Supplementary File [file srep42813-s1.pdf]

Preventive Effects of Carnosine on Lipopolysaccharide-induced Lung Injury

KEN-ICHIRO TANAKA[1], TOSHIFUMI SUGIZAKI[2], YUKI KANDA[1], FUMIYA TAMURA[1], TOMOMI NIINO[1] and MASAHIRO KAWAHARA[1]

[1]Laboratory of Bio-Analytical Chemistry, Research Institute of Pharmaceutical Sciences, Musashino University, 1-1-20 Shinmachi, Nishitokyo-shi, Tokyo 202-8585, Japan.

[2]Department of System Chemotherapy and Molecular Sciences, Division of Bioinformatics and Chemical Genomics, Graduate School of Pharmaceutical Sciences, Kyoto University, Sakyo-ku, Kyoto 606-8501, Japan.

Correspondence

Dr. Ken-ichiro Tanaka, Laboratory of Bio-Analytical Chemistry, Research Institute of Pharmaceutical Sciences, Musashino University, 1-1-20 Shinmachi, Nishitokyo-shi, Tokyo 202-8585, Japan. TEL & FAX: 81-42-468-9335, E-mail: k-tana@musashino-u.ac.jp

Running head

Carnosine and Acute Lung Injury

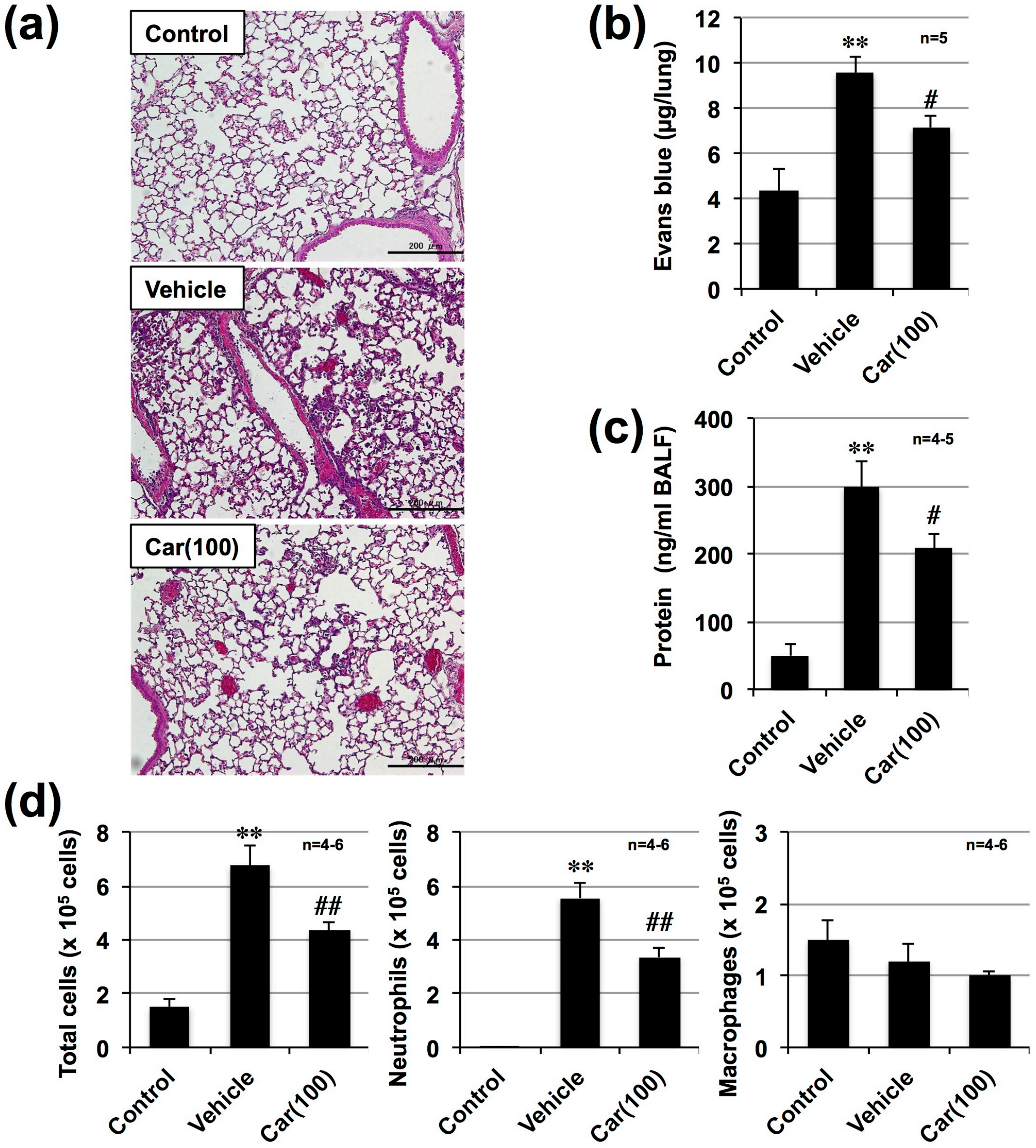

Fig. S1. Effect of carnosine on zymosan-induced lung injury. Male ICR mice were intratracheally administered with zymosan (1 mg/kg) or the zymosan vehicle (Control). Mice were orally administered with carnosine (Car, 100 mg/kg) or saline (Vehicle) immediately prior to zymosan administration. Sections of pulmonary tissue (a) or BALF (c, d) were prepared 24 h after zymosan administration. Sections were subjected to histopathological examination (H&E staining) (scale bar, 200 μm) (a). Evans blue dye (30 mg/kg) was administered intravenously 6 h after zymosan administration, and 2 h later, was extracted from the lung samples and quantified (b). The amount of protein present in the BALF was determined by the Bradford method (c). The numbers of total cells, neutrophils and macrophages in BALF were determined (d). Values are mean ± S.E.M.; $^{\#}P$<0.05; ** or $^{\#\#}P$<0.01 (*, vs Control; #, vs Vehicle).

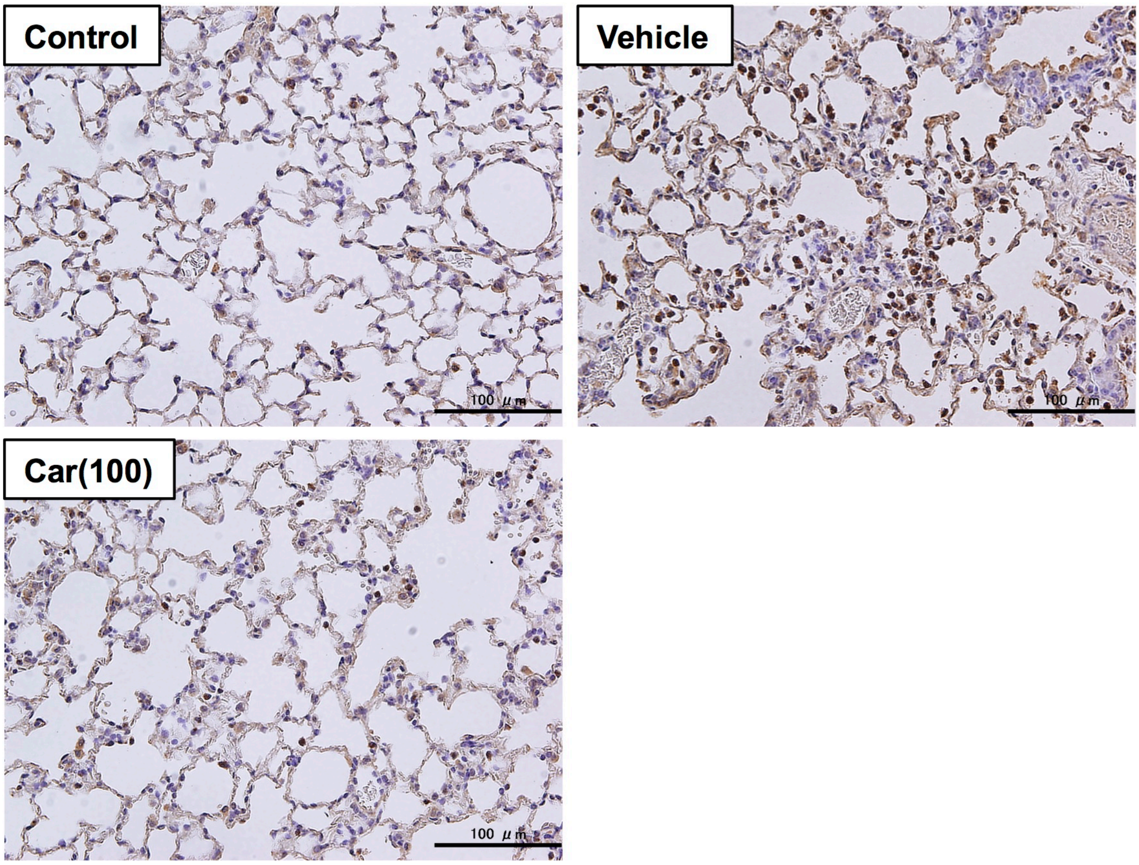

Fig. S2. Effect of carnosine on LPS-induced neutrophil elastase expression. Male ICR mice were intratracheally administered with LPS (1 mg/kg) or LPS vehicle (Control). Mice were orally administered with carnosine (Car, 100 mg/kg) or saline (Vehicle) immediately before and 24 h after LPS administration. Sections of pulmonary tissue were prepared 48 h after LPS administration. Immunohistochemical analysis of pulmonary tissue was performed with an antibody against neutrophil elastase (scale bar, 100 μm).

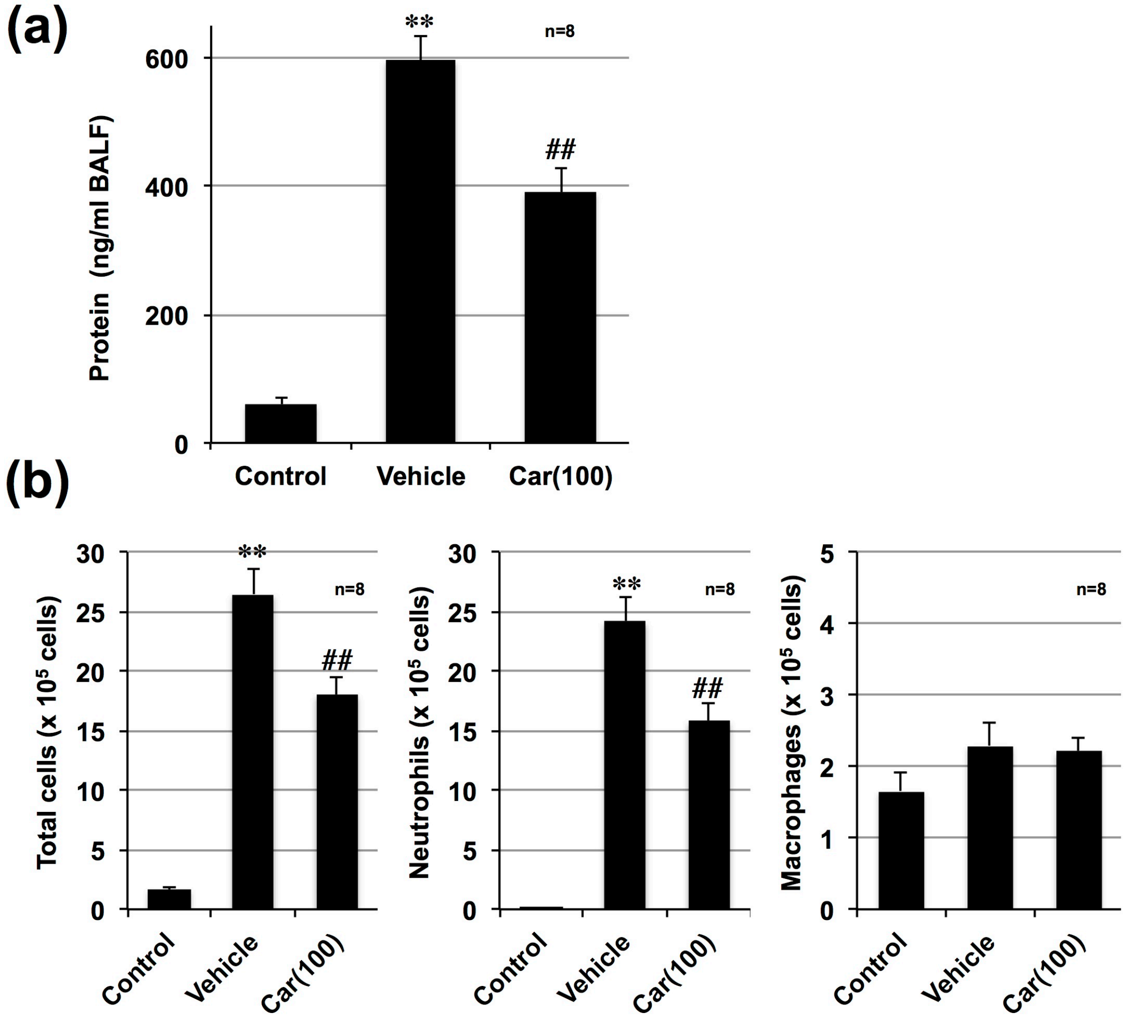

Fig. S3. Effect of carnosine administration after the onset of LPS-induced lung injury. Male ICR mice were intratracheally administered with LPS (1 mg/kg) or LPS vehicle (Control). Mice were orally administered with carnosine (Car, mg/kg) or saline (Vehicle), 1 h and 24 h after LPS administration. BALF were prepared 48 h after LPS administration. The amount of protein present in the BALF was determined by the Bradford method (a). The numbers of total cells, neutrophils and macrophages in BALF were determined (b). Values are mean ± S.E.M.; ** or $^{##}P$<0.01. (*, vs Control; #, vs Vehicle).

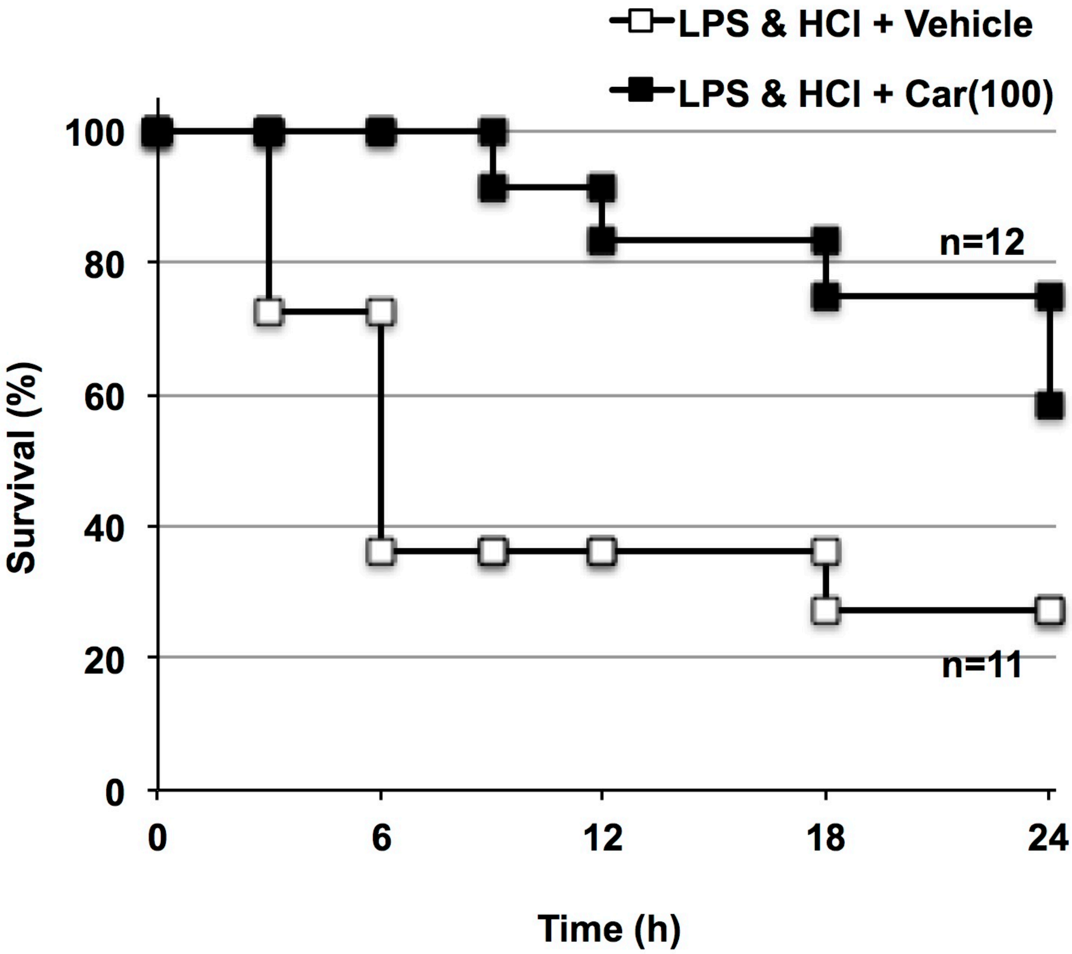

Fig. S4. Effect of carnosine on the survival of mice subjected to LPS and hydrochloric acid (HCl). Male ICR mice were orally administered with carnosine (Car; 100 mg/kg) or saline (Vehicle). Then, mice were intratracheally administered with HCl (0.2 M, 2 ml/kg). One hour after the HCl administration, mice were intratracheally administered LPS (40 mg/kg). Survival was monitored at 3, 6, 9, 12, 18 and 24 h after the LPS administration.

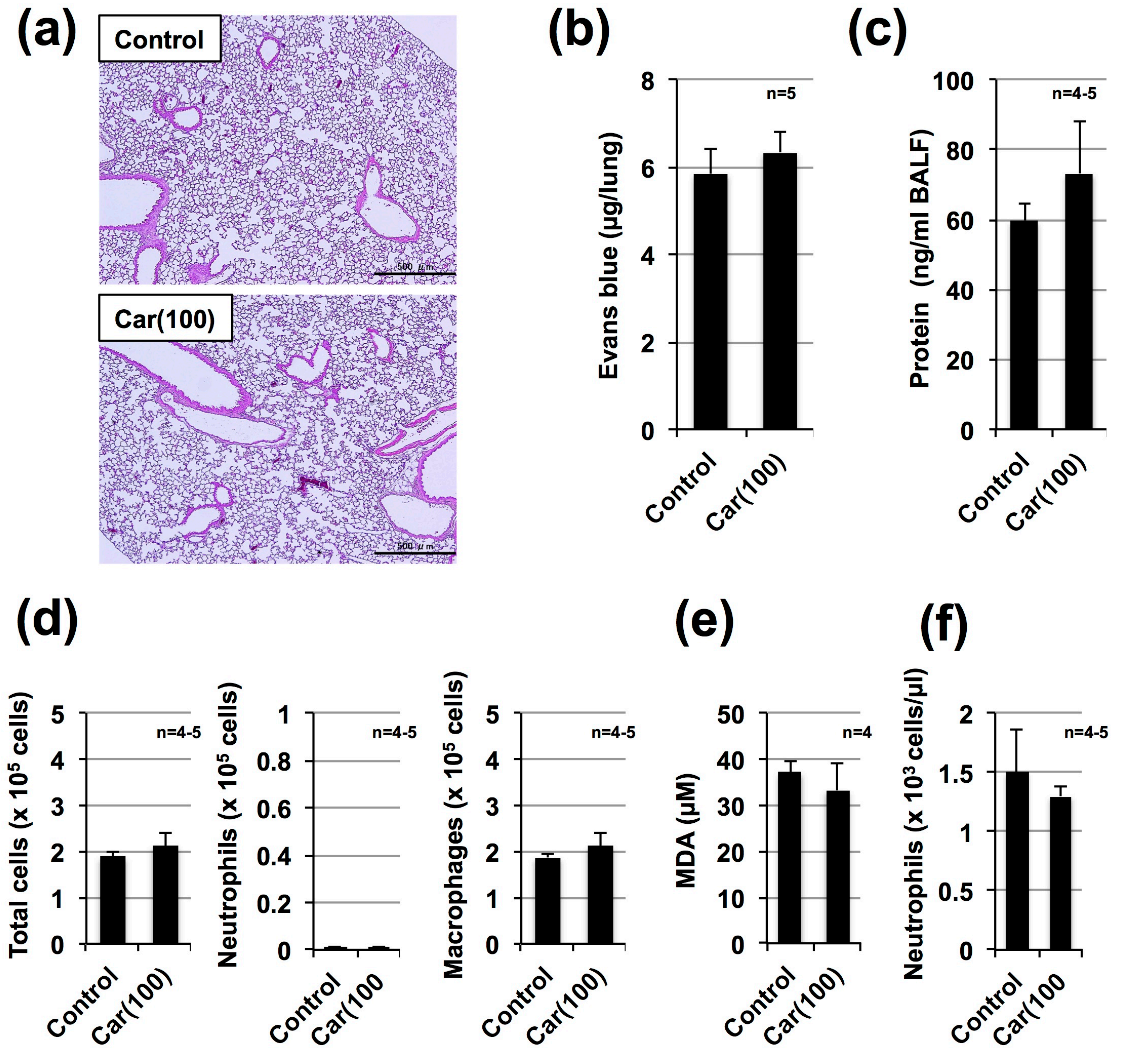

Fig. S5. Effect of administering carnosine alone. Mice were orally administered with carnosine (Car, 100 mg/kg) or saline (Control). Sections of pulmonary tissue (a), BALF (c, d) or plasma (e) were prepared 24 h after carnosine administration. Sections were subjected to histopathological examination (H&E staining) (scale bar, 500 µm) (a). Evans blue dye (30 mg/kg) was administered intravenously 6 h after carnosine administration, and 2 h later, was extracted from the lung samples and quantified (b). The amount of protein present in the BALF was determined by the Bradford method (c). The numbers of total cells, neutrophils and macrophages in BALF were determined (d). The levels of malondialdehyde (MDA) in the plasma were determined using TBARS Assay Kit (e). Whole blood was prepared 1 h after carnosine administration. The number of neutrophils in the blood was determined using an automated haematology analyzer (f). Values are mean ± S.E.M.
